# Revision of *Claraeola* (Diptera, Pipunculidae) in the Middle East based on morphology and DNA barcodes

**DOI:** 10.3897/zookeys.873.36645

**Published:** 2019-08-29

**Authors:** Behnam Motamedinia, Jeffrey H. Skevington, Scott Kelso

**Affiliations:** 1 Canadian National Collection of Insects, Arachnids and Nematodes, Agriculture and Agri-Food Canada, 960 Carling Avenue, Ottawa, ON K1A 0C6, Canada Agriculture and Agri-Food Canada Ottawa Canada; 2 Plant Protection Research Department, South Khorasan Agricultural and Natural Resources Research and Education Center, AREEO, Birjand, Iran South Khorasan Agricultural and Natural Resources Research and Education Center Birjand Iran

**Keywords:** big-headed flies, COI, distribution map, DNA barcoding, identification key, mini barcode protocol

## Abstract

The Middle East species of *Claraeola* Aczél (Diptera, Pipunculidae) are revised based on morphological characteristics and sequence data from the mitochondrial COI barcoding gene, using a novel COI mini-barcode protocol. Four new *Claraeola* species are described: *C.bousynterga* Motamedinia & Skevington, **sp. nov.**, *C.heidiae* Motamedinia & Skevington, **sp. nov.**, *C.khuzestanensis* Motamedinia & Skevington, **sp. nov.**, and *C.mantisphalliga* Motamedinia & Skevington, **sp. nov.***Eudorylasthekkadiensis* Kapoor, Grewal & Sharma, 1987 is transferred to *Claraeola*, *C.thekkadiensis* (**comb. nov.**). Diagnoses, illustrations, an identification key, and a distributional map are given for the Middle East species.

## Introduction

Pipunculidae Walker 1834 (Diptera), commonly known as big-headed flies, are important endoparasitoids of Auchenorrhyncha (Hemiptera), particularly the families Cicadellidae, Delphacidae and Cercopidae, and Tipulidae (Diptera) (Koenig and Young 2007; Rafael and Skevington 2010). Females of big-headed flies typically lay only one egg inside each host and rates of parasitism vary from a fraction of a percent to 100% in local populations (Skevington and Marshall 1997). Since many species of Auchenorrhyncha are known to transmit plant diseases (Weintraub and Beanland 2006), Pipunculidae have the potential of becoming biological control agents of economically important pest species such as green rice leafhoppers, *Nephotettix* spp. feeding on rice, or the potato leafhopper, *Empoascafabae* (Harris, 1841), which severely damages potato and alfalfa crops (Hardy 1964; Jervis 1992). Pipunculidae are characterized by large compound eyes that occupy most of their hemispherical head, distinctive wing venation, the piercer-like shape of the female ovipositor, and the presence of a chitinized postspiracular plate in the last instar larvae of the subfamilies Chalarinae and Pipunculinae (Wada 1991; Rafael and De Meyer 1992). Currently, 1,450 species of pipunculids are recognized worldwide, placed into four subfamilies and 20 genera (Kehlmaier et al. 2014; Kehlmaier et al. 2019; Skevington 2019). The genus *Claraeola* Aczél, 1940 comprises 35 described species, occurring in the Afrotropical (four species; Földvári 2013), Palaearctic (17 species; Kehlmaier 2005a; Kehlmaier 2005b), Oriental (seven species; Kozánek and Kwon 1991; Kozánek et al. 2003, Kehlmaier 2005b), and Australasian (seven species; Skevington 2002) regions. Placed within the diverse tribe Eudorylini, *Claraeola* can best be separated from the other genera by the hind tibia having a mid-anteriorly wrinkled indentation bearing some erect setae and the presence of at least some small but distinct tooth-like projections on the male phallus, which are arranged either on a membranous sheath or on the ejaculatory ducts itself (Kehlmaier 2005a; Motamedinia et al. 2017b). Aczél (1940) erected *Claraeola* from one previously described species, *Dorylasadventitius* Kertész, 1912. This genus has been re-defined by Skevington and Yeates (2001) and found to be senior synonym of *Congomyia* Hardy, 1949 and *Moriparia* Kozánek & Kwon, 1991. Skevington (2002) revised the Australian species of *Claraeola*, whereas Kehlmaier (2005a, 2005b) and Földvári (2013) revised the Palaearctic/Oriental and Afrotropical members of this genus, respectively, but there is no information about its biology or immature stages.

The Middle East [here defined to include Bahrain, Cyprus, Iran, Iraq, Israel, Jordan, Kuwait, Lebanon, Oman, Palestine, Qatar, Saudi Arabia, Syria, Turkey, United Arab Emirates, and Yemen] is located between three zoogeographic realms (Palaearctic, Oriental, and Afrotropical regions). However, only four species of *Claraeola* [*C.halterata* (Meigen, 1838); *C.conjuncta* (Collin, 1949); *C.parnianae* Motamedinia & Kehlmaier, 2017, and *C.khorshidae* Motamedinia & Kehlmaier, 2017] have been previously reported from this region (Kehlmaier 2005a, 2005b; Motamedinia et al. 2017a, 2017b). As recently studied material brought to light additional unnamed species of the genus, the purpose of this work is to revise *Claraeola* species from the Middle East region. The revision includes descriptions of four new species, photo illustrations of important morphological characters, a distribution map and an identification key to the Middle Eastern *Claraeola* species.

## Materials and methods

### Insect material

The study is based on material deposited in the Canadian National Collection of Insects, Arachnids and Nematodes (**CNC**; Ottawa, Canada), the Hayk Mirzayans Insect Museum (**HMIM**; Insect Taxonomy Research Department, Iranian Research Institute of Plant Protection, Tehran, Iran, (Senckenberg Natural History collections Dresden (**SMTD**; Dresden, Germany); Indian Agriculture Research Institute (**INPC**; New Delhi, Inida), and the Tel Aviv University (**TAU**; Tel Aviv, Israel). The specimens were collected using Malaise traps, sweep nets, and pan traps. Full descriptions are presented for new species only, and a brief diagnosis is provided for known species. Most male genitalia were separated from the abdomen, heated in lactic acid (85%) at 100 °C for 30–240 minutes, and then placed into a drop of glycerin on a microscope slide. Potassium hydroxide was used for terminalia that were very darkly pigmented or that were to be used for photography. For this, terminalia were treated with 10% KOH at 100 °C for 10–30 minutes then immersed in glacial acetic acid for 5 minutes to buffer the reaction and stop the clearing. Following clearing, dissection involved separating syntergosternite 8 and the epandrium from the remainder of the abdomen. For photography, the epandrium was removed to fully expose the hypandrium and phallic structures. The dissected genitalia are stored in plastic microvials with glycerin on the same pin as the source specimen. All specimens are labeled with a unique reference number from the CNC database (e.g., Jeff_Skevington_Specimen12345 and CNC_Diptera12345, abbreviated as JSS12345 and CD12345 respectively) and can be accessed at https://cnc.agr.gc.ca/. Species are described in alphabetical order. SimpleMappr (Shorthouse 2010) was used to create the species distribution map.

External characters were imaged using a Leica DFC450 module fitted on a Leica M205C stereomicroscope using 0.6× lens. Final images were merged using the image-stacking software Zerene Stacker (Littlefield 2018). Images of the genitalia were taken using a Leica DM5500B microscope equipped with a Leica DMC4500 module connected to a personal computer running the Leica Application Suite software (https://www.leica-microsystems.com), which includes an Auto-Montage module that combines multiple layers of photographs into a single fully focused image. All photos were subsequently modified using Adobe Photoshop CS3 imaging software. The morphological terminology follows Skevington (2002) and Kehlmaier (2005a) with the following abbreviations being used throughout the paper:

**LF:WF** ratio of length of flagellum to its width.

**LW:MWW** ratio of length of wing to maximum width of wing.

**LS:LTC** ratio of length of pterostigma to length of third costal segment.

**LTC:LFC** ratio of length of third costal segment to length of fourth costal segment.

**LT35:WT5** ratio of length of tergites 3–5 to maximum width of tergite 5.

**WT5:LT5** ratio of width of tergite 5 to its length.

**T5R:T5L** ratio of length of right margin of tergite 5 to length of its left margin.

**LT35:WS8** ratio of length of tergites 3–5 to width of syntergosternite 8.

**LS8:HS8** ratio of length syntergosternite 8 to its height.

**MLE:MWE** ratio of maximum length of epandrium to its maximum width (viewed dorsally).

**LP:LB** ratio of length of piercer to length of base (viewed laterally).

**LDP:LPP** ratio of length of distal part of piercer to length of its proximal part (viewed laterally).

### DNA extraction, PCR amplification, and sequencing

Total genomic DNA was extracted either from two legs or from whole specimens using the DNeasy Blood and Tissue Kit (Qiagen Inc., Santa Clara, CA, USA) following the manufacturer’s protocol. Following extraction, specimens were critical-point dried and deposited as vouchers in the CNC.

For DNA barcoding, a 658 bp fragment of the 5' end of the mitochondrial coding gene cytochrome oxidase subunit I (COI) was amplified using the primer pair LCO1490 and COI-Dipt-2183R, as previously described by Gibson et al. (2011). In some cases, initial attempts to amplify the full COI barcode failed, presumably due to the degradation of the DNA. In these cases, a novel COI mini-barcode protocol was employed (Young et al. in prep.) in order to amplify a 214 bp fragment (COI-Fx-C), located at the3'-end of the COI barcode region, for species identification. In the case of putative new species, efforts were made to amplify the5' and middle COI mini-barcode fragments (COI-Fx-A and COI-Fx-B respectively) that, when combined, provide a complete COI barcode sequence. Oligonucleotides (primers) used in this study are listed in Table 1. PCR amplifications were carried out in 25μl volumes, including 15.7μl ddH_2_O, 2.5μl 10X Ex Taq PCR buffer (containing 20mM MgCl_2_), 0.65μl 25mM MgCl_2_, 1μl of each 10μM primer, 2μl 10mM dNTPs, 0.15μl Ex Taq HS DNA polymerase (TaKaRa Bio USA, Madison, WI, USA), and 2μl total DNA. Amplification cycles were performed on an Eppendorf ep Gradient S Mastercycler (Eppendorf AG, Hamburg, Germany). All PCR and sequencing reactions were performed with the following thermal cycler conditions: 94 °C for 3 mins × 1 cycle, 94 °C for 45 secs, 45 °C for 45 secs, 72 °C for 1 min × 45 cycles, 72 °C for 5 minutes × 1 cycle, followed by an unlimited step at 10 °C. Amplification products were visualized on 1% agarose electrophoresis gels and purified prior to sequencing using either Clone-Well 0.8% Egels (Invitrogen™, Carlsbad, CA, USA) for full barcode amplicons, or an ExoSAP-IT protocol (USB Corp., Cleveland, OH, USA) for COI-Fx amplicons. Sequencing reactions were carried out in 10μl volumes, using the ABI BigDye Terminator v3.1 Cycle Sequencing kit (PE Applied Biosystems, Foster City, CA, USA). Bidirectional sequencing reactions were purified using the ABI ethanol/EDTA/sodium acetate precipitation protocol and analyzed on an ABI 3500xl Genetic Analyzer (PE Applied Biosystems, Foster City, CA, USA). Sanger Sequencing was performed at CNC.

**Table 1.** Cytochrome c oxidase subunit I mitochondrial gene primers.

Gene name/region	Forward primer name	Forward primer sequence (5’-3’)	Primer reference	Reverse primer name	Reverse primer sequence (5’-3’)	Primer reference
COI Barcode	LCO1490	GGTCAACA	Folmer et al. (1994)	COI-Dipt-2183R	CCAAAAAATC	Gibson et al. (2011)
		AATCATAAA			ARAATARRTG	
		GATATTGG			YTG	
COI-Fx-A (5’ end of barcode)	LCO1490	GGTCAACA	Folmer et al. (1994)	COI-SYR-1762R	CGDGGRAAD	Young et al. (in prep.)
		AATCATAAA			GCYATRTCDGG	
		GATATTGG				
COI-Fx-B (middle of barcode)	COI-SYR-342F	GGDKCHCC	Young et al. (in prep.)	COI-SYR-1976R	GWAATRAART	Young et al. (in prep.)
		NGAYATRGC			TWACDGCHCC	
COI-Fx-C (3’ end of barcode)	COI-SYR-1957F	GGDATWTC	Young et al. (in prep.)	COI-Dipt-2183R	CCAAAAAATCA	Gibson et al. (2011)
		HTCHATYYTAGG			RAATARRTGYTG	

All sequence chromatograms were edited and contigs formed using Sequencher 5.4.6 (Gene Codes Corp., Ann Arbor, MI, USA). Resulting contigs were hand-aligned using Mesquite 3.6 (Maddison and Maddison 2018). Uncorrected pairwise genetic distances (p-distance) were calculated with Mega7 (Kumar et al. 2016). Sequence accession numbers issued by GenBank (**GB**) and the European Nucleotide Archive (**ENA**) are provided for each specimen.

## Results

### Key to males of *Claraeola* species in the Middle East

**Table:** 

1	Abdominal tergites with narrow but distinct yellow markings (Fig. 9F)	**2**
–	Abdominal tergites without narrow yellow markings (Fig. 9A–D)	**3**
2	Mid and hind tibiae with erect anteromedial setae (Fig. 9E); ejaculatory ducts with small teeth (Fig. 5D)	***C.khuzestanensis* sp. nov.**
–	Only hind tibia with erect anteromedial setae; ejaculatory ducts without small teeth (Fig. 7D, E)	***C.parnianae* Motamedinia & Kehlmaier**
3	Phallus embedded in membranous sheath (Figs 2D, 6D)	**4**
–	Phallus without distinctly membranous sheath	**7**
4	Legs light-brown (Fig. 9B); surstyli with enlarged separation at base (Fig. 6D)	***C.mantisphalliga* sp.nov.**
–	Legs dark (Fig. 9D); surstyli without enlarged separation at base	**5**
5	Abdominal tergite 2 with some long setae laterally (Fig. 9C); surstyli in lateral view with humped base dorsally (Fig. 3D, E); membranous sheath surrounding ejaculatory ducts weak with small teeth (Fig. 3E)	***C.halterata* (Meigen)**
–	Abdominal tergite 2 without some long setae laterally; genitalia not as above	**6**
6	Syntergosternite 8 large (Fig. 9A); left gonopod longer than right one (Fig. 1B); surstyli as in Fig. 1C–D	***C.bousynterga* sp. nov.**
–	Syntergosternite 8 small; left gonopod as long as right one (Fig. 2B); surstyli as in Fig. 2D, E	***C.conjuncta* (Collin)**
7	Surstyli straight in lateral view; ejaculatory ducts with small spines (Fig. 4D, E)	***C.heidiae* sp. nov.**
–	Surstyli bent in lateral view; ejaculatory ducts with large spines (Fig. 11B, F, G)	***C.khorshidae* Motamedinia & Kehlmaier**

#### Claraeola

Taxon classificationAnimaliaDipteraPipunculidae

Aczél, 1940

30B8CE1AF61B5B148443C59292BCC4FC

Claraeola Aczél, 1940: 151. Type species: Dorylasadventitius Kertész, 1912, by original designation.
Congomyia Hardy, 1949b: 7. Syn. by Skevington and Yeates 2001: 429. Type species: Congomyianigripennis Hardy, 1949, by original designation. Syn. by Skevington and Yeates 2001: 429.
Moriparia Kozánek & Kwon, 1991: 77. Type species: Moriparianigripennis Kozánek & Kwon, 1991, by original designation. Syn.: Skevington and Yeates 2001: 429.

##### Diagnosis.

Medium to large big-headed flies, body length 3.2‒7.4 mm, wing length 3.2‒8.4 mm, pedicel with 4‒10 upper and 3‒10 lower bristles, flagellum gray to brownish gray pruinose, frons silver-gray pruinose with a weak median keel, postpronotal lobe with 6‒18 setae, scutellum with 8‒22 short setae along posterior margin, hind tibia with a wrinkled indentation mid-anteriorly bearing some erect setae, pterostigma present, cross-vein r-m reaches dm at or after one third of the cells length, abdomen ovate or elongate, ground color dark (in some specimens with narrow posterolateral yellow marks), tergite 1 with 3–20 long bristles, situated in one to three rows, tergite 2 with or without lateral bristles, membranous area medium to large, epandrium mostly wider than long (LS8:HS8 < 1), phallus partly clothed in small, but distinct setae or teeth, arranged on membranous sheath or on ejaculatory ducts.

##### Biology.

Unknown

##### Distribution.

Palearctic (Algeria, Austria, Belgium, Canary Islands, China, Cyprus, Czech Republic, Denmark, Egypt, France, Germany, Great Britain, Greece, Hungary, Iran, Israel, Italy, Latvia, Lithuania, Netherlands, North Korea, Russia, Slovakia, South Korea, Sweden, Switzerland, Tunisia), Oriental (Borneo, India, Myanmar, Nepal, Philippines, Taiwan, Thailand, Vietnam), Afrotropical (Burundi, Cameroon, Congo, Madagascar, Malawi, Uganda), and Australian (Australia, Papua New Guinea) (Skevington and Yeates 2001; Skevington 2002; Kehlmaier 2005a, 2005b; Földvári 2013; Motamedinia et al. 2017a, 2017b; Kehlmaier et al. 2019).

### Taxonomic treatment of species, in alphabetical order

#### Claraeola bousynterga

Taxon classificationAnimaliaDipteraPipunculidae

Motamedinia & Skevington sp. nov.

876793D96C7756F491074BDB052EF0D5

http://zoobank.org/7AB2F6DF-3AFC-4B73-A142-0E0B3537DC68

Figs 1 , 8A , 9A

##### Examined material.

***Holotype.*** IRAN • ♂; Sistan & Balochestan, Saravan; 27°25'N, 62°17'E; 8 Nov. 2016; F. Hamzavi leg.; pan trap; JSS51920; GB: MN182733; CNC. ***Paratypes.*** IRAN • 1 ♂; same data as holotype; JSS51829; GB: MN182745; CNC • 1 ♀; same data as holotype; 2 Sep. 2015; sweep net; JSS52173; GB: MN182734; CNC.

##### Diagnosis.

Due to the shape of the surstyli, phallus and phallic guide, this species is related to the *clavata* species group: *C.discors* (Hardy, 1966), known from Nepal and partly illustrated by Kehlmaier (2005b), *C.clavata* (Becker, 1897), known from Europe and re-described by Kehlmaier (2005a), *C.conjuncta* (Collin, 1949), *C.khorshidae* Motamedinia & Kehlmaier, 2017 and *C.thekkadiensis* (Kapoor, Grewal & Sharma, 1987) comb. nov. It differs from these species by the shape of surstyli in lateral view being weakly bent, broad syntergosternite 8 and a large membranous area.

##### Description.

**Figure 1.**
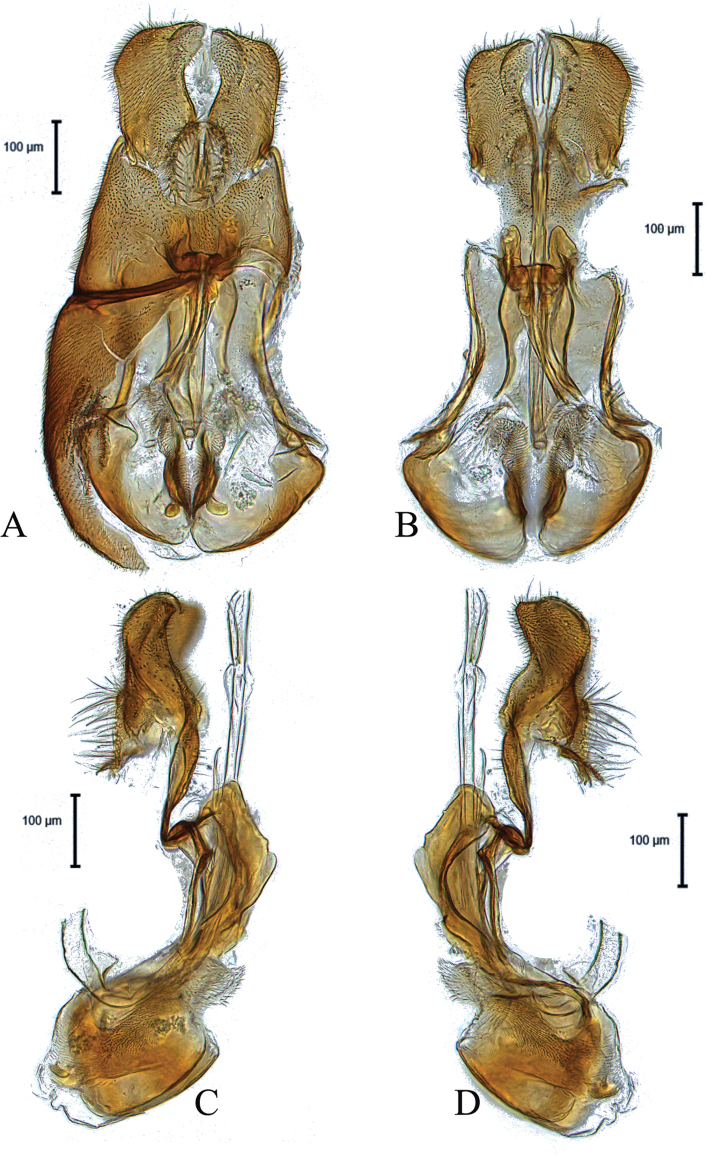
Male genitalia of holotype *Claraeolabousynterga* Motamedinia & Skevington, sp. nov. **A** in dorsal view **B** in ventral view **C, D** in lateral view.

**Male.** Body length (excluding antennae): 4.2–4.3 mm (n = 2). ***Head.*** Scape, pedicel and arista dark brown, pedicel with a pair of short plus long upper and lower bristles, lower bristles longer than upper bristles, flagellum tapering and light brown pruinose (LF:WF = 2.2); arista with thickened base. Eyes meeting for a distance of 16 facets. Frons dark silver-gray pruinose. Vertex black, bearing an elevated slightly ocellar triangle. Occiput dark and gray pruinose with a row of long setae along posterior margin. ***Thorax.*** Postpronotal lobe dark, gray pruinose. Prescutum and scutum black with scattered long setae at anterior supra-alar area. Scutellum black with ca. 12 thin short setae along posterior margin (up to 0.05 mm). Subscutellum black, gray pruinose. Pleura dark brown ***Wing.*** Length: 3.5–3.8 mm. LW:MWW = 3.0. Wing almost entirely covered with microtrichia. Pterostigma dark-brown and complete. LS:LTC = 1.0. LTC:LFC = 1.5. Cross-vein r-m reaches dm shortly after one third of the cell’s length. M_1_ strongly undulating in middle. Halter length: 0.7 mm, base and knob dark, stem narrowly white or dark. ***Legs.*** Coxae dark, gray pruinose. Mid coxa with two or three black anterior bristles. Trochanters partly gray pruinose, mid trochanter with two or three black anterior bristles, hind trochanter partly yellow with 4–6 brown anterior bristles. Femora dark with pale apices, gray pruinose with two or three wrinkled indentations at base. Mid and hind femora bearing two rows of dark anteroventral small spines in apical half. Tibiae gray pruinose, with two rows of short setae on anterior side and three rows on posterior. Hind tibia with two or three wrinkled indentations in middle without erect anteromedial setae. Tarsi yellowish at posterior margin but darkened with scattered dark setae at anterior margin. Pulvilli yellow. Claws brown with black tips. ***Abdomen.*** Ground color dark, tergite 1 silver-gray pruinose, with three or four dark lateral bristles (up to 0.1 mm). Tergites 2–5 posterolaterally gray pruinose, slightly extending onto dorsal surface along posterior margin, largest on tergite 5 where they extend onto dorsal surface, otherwise brown pruinose. Tergite 5 slightly longer than tergite 4 and almost symmetrical in dorsal view (LT35:WT5 = 1.1, WT5:LT5 = 0.6, T5R:T5L = 1.0). Sternites white-yellow laterally and brown with dark mid-line centrally, gray pruinose. Syntergosternite 8 enlarged, dark brown and gray pruinose without dorsal depression on side of right surstylus. LT35:WS8 = 2.5. Viewed laterally, longer than high (LS8:HS8 = 1.8). Membranous area large and roundish, more than one third of the width of syntergosternite 8. ***Genitalia.*** Genital capsule in dorsal view: epandrium and surstyli dark brown, inner side of both surstyli yellow, gray pruinose. Epandrium wider than long (MLE:MWE = 0.53). Surstyli rather symmetrical. Left surstylus slightly smaller than right one, right surstylus with slightly broadened tip (Fig. 1A). Genital capsule in ventral view: subepandrial sclerite wide with scattered setae, gonopod medium sized with two projections in its middle, inner gonopod slightly higher than outer one (Fig. 1B). Genital capsule in lateral view: phallus straight, strong and long, with three short ejaculatory ducts, two of them bearing small teeth along their sides; both surstyli slightly narrowed in middle, right surstylus slightly larger than left surstylus (Fig. 1C, D). Phallic guide small, reaching base of surstyli. Epandrium without projecting lobe on either side. Genital capsule in dorsal view: surstyli rather rectangular, base and tips broadened (Fig. 1A).

**Female.** Body length (excluding antennae): 3.6mm (n = 1). Eyes separated. Frons gray pruinose. Occiput gray pruinose. Postpronotal lobe dark, yellow in upper margin, gray pruinose. Scutum black, gray pruinose with scattered setae at anterior supra-alar area. Wing length: 3.7 mm. LW:MWW = 2.4. Pterostigma light-brown and slightly complete (LS:LTC = 0.98, LTC:LFC = 0.9). Mid coxa with 3–5 black anterior bristles. Femora bearing two small ventral rows of dark peg-like spines in the apical third. Hind tibia without distinctly stronger bristly setae. Tergites 1–2 gray pruinose, tergites 3–5 posterolaterally gray pruinose, slightly extending onto dorsal surface along posterior margin, otherwise brown pruinose. ***Ovipositor.*** Viewed laterally: piercer long (LP = 0.7 mm), strongly curved upward and reaching sternite 2 (Fig. 8A). LP:LB=2.3. LDP:LPP = 1.8.

##### Etymology.

From prefix *bou* meaning large in Greek and *synterg* (syntergosternite 8), referring to a large syntergosternite 8.

##### Distribution.

Iran (Fig. 10).

#### Claraeola conjuncta

Taxon classificationAnimaliaDipteraPipunculidae

(Collin, 1949)

C42D50AB691E5D8A95FB801DBB81AB4E

Fig. 2

Eudorylas conjunctus Collin 1949: 191.

##### Examined material.

ISRAEL • 2♂♂; Hazeva Field School; 30°43'N, 35°15'E; 21 Jan. 1997, A. Maklakov leg.; Malaise trap; JSS50791; JSS50784; GB: MN182738; TAU • 1♀; 10 Oct. 1997, A. Maklakov leg.; Malaise trap; JSS51646; TAU • 1♀; 12 Dec. 1997; A. Maklakov leg.; Malaise trap; JSS51647; TAU • 1♂; 30°43'N, 35°15'E; 3 Oct. 1997, A. Maklakov leg.; Malaise trap; JSS51705; TAU • 1♂; Qalya; 28 Sep. 1995; A. Freidberg leg.; JSS50783; TAU • 1♂; Zomet Qetura; 29°59'N, 35°4'E; 15 May 2010; A. Freidberg leg.; JSS50804; TAU • 1♀; Ne’ot Semadar; 30°43'N, 35°15'E; 4 Dec. 1995; A. Freidberg leg.; JSS51649; TAU.

##### Diagnosis.

**Figure 2.**
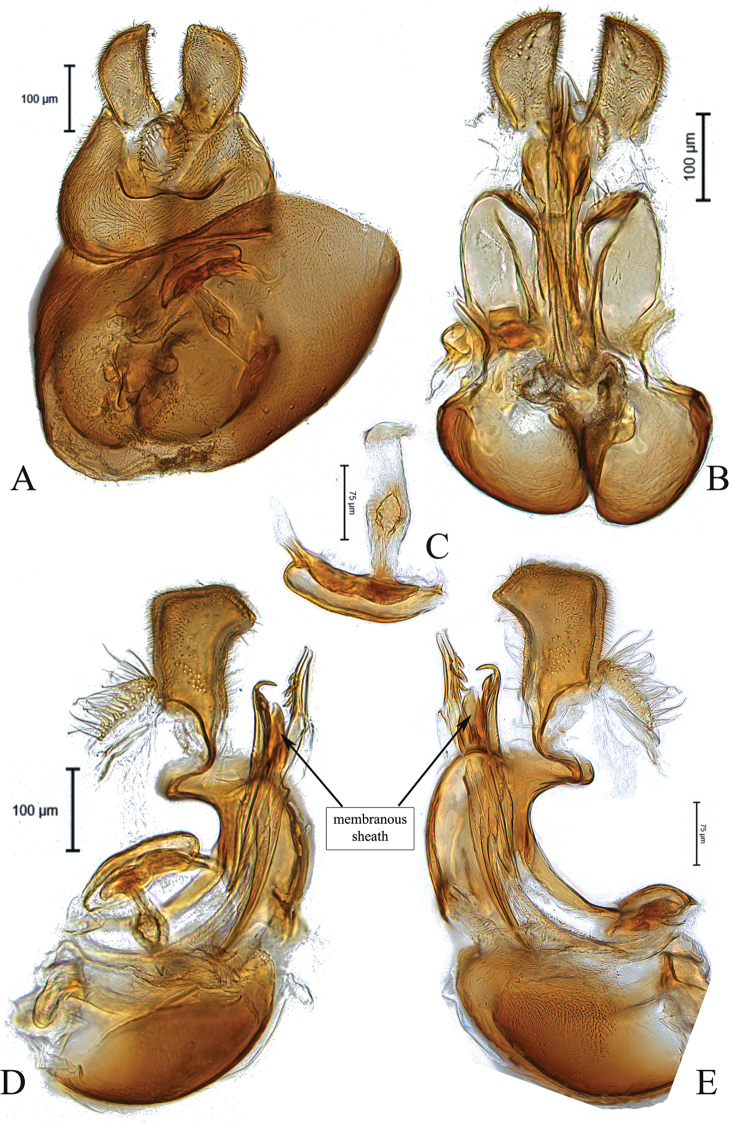
Male genitalia of *Claraeolaconjuncta* (Collin) **A** in dorsal view **B** in ventral view **C** ejaculatory apodeme **D, E** in lateral view.

This species can be recognized by large ventral spines on the hind femur, shining on basal half of front and mid femora ventrally; surstyli slightly bow-shaped in dorsal view (Fig. 2A), each gonopod with six to seven strong bristles on inner side (Fig. 2B); phallus with three broad and slightly bent ejaculatory ducts, two of them bearing two to three saw-like teeth along their side; phallic guide short but broad, especially at apex (Fig. 2D, E).

##### Distribution.

Algeria, Egypt, Israel (Fig. 10), Tunisia (Kehlmaier 2005b; Kehlmaier et al. 2019).

#### Claraeola halterata

Taxon classificationAnimaliaDipteraPipunculidae

(Meigen, 1838)

519A2D882DDA5B05B73D053DA8D4C704

Figs 3 , 9C–D

Pipunculus halteratus Meigen 1838: 146.

##### Examined material.

ISRAEL • 1♂; Har Hermon; 31°46'N, 34°37'E; 11 Jun. 2003; A. Freidberg leg.; JSS51645; GB: MN182742; TAU.

##### Diagnosis.

**Figure 3.**
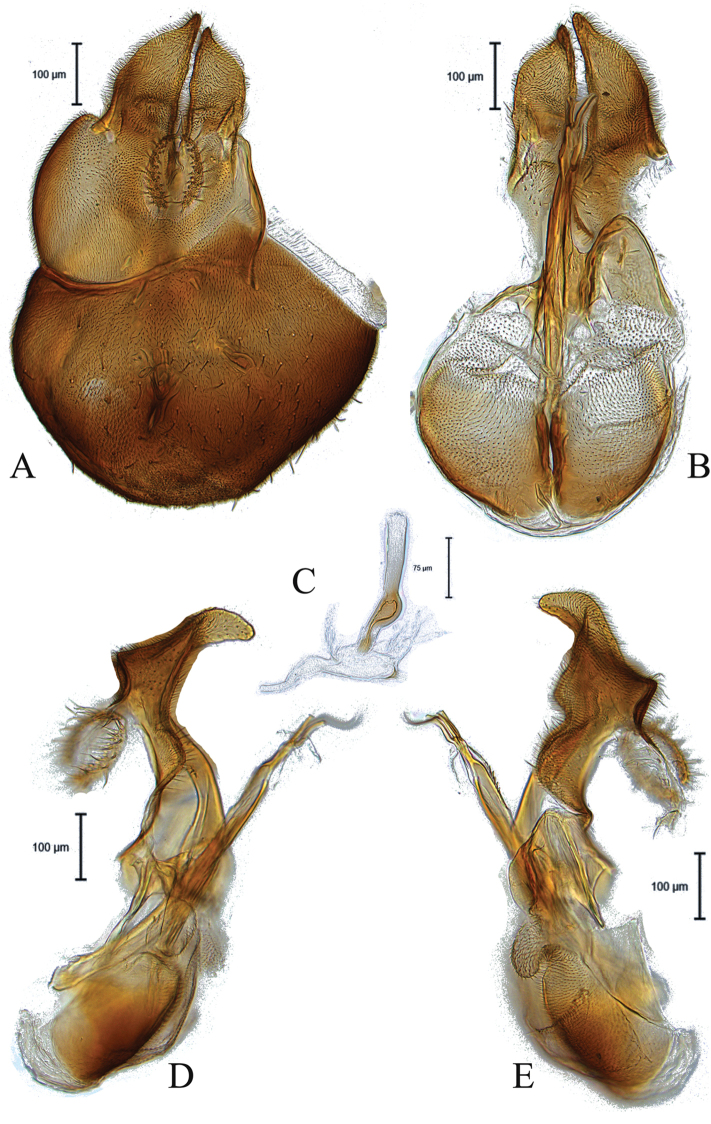
Male genitalia of *Claraeolahalterata* (Meigen) **A** in dorsal view **B** in ventral view **C** ejaculatory apodeme **D, E** in lateral view.

This species can be recognized by dark legs; surstyli in lateral view with dorsally humped base (Fig. 3D, E); large outer gonopod (Fig. 3B).

##### Distribution.

Austria, Belgium, France (mainland), Germany, Great Britain, Hungary, Israel (Fig. 10), Latvia, Netherlands, Slovakia (Kehlmaier 2005a).

##### Remarks.

Meigen (1838) described the species from Belgium. Due to the loss of all type material, Kehlmaier (2005a) designated a neotype and redescribed the species. The neotype is from Vernditch (southern England) and is deposited in The Natural History Museum, London (England). This species was examined by Skevington and Yeates (2001) for their phylogenetic study of Eudorylini.

#### Claraeola heidiae

Taxon classificationAnimaliaDipteraPipunculidae

Motamedinia & Skevington sp. nov.

3D14FE87F41A52C5883C1D25CCBA9331

http://zoobank.org/F3D2A78C-082F-4EF3-BAAB-752978DDFF1E

Fig. 4

##### Examined material.

***Holotype.*** YEMEN • ♂; Manakhah; 15°04'N, 43°44'E; 6 Jul.–21 Aug. 2002; Malaise trap; A. van Harten leg.; CD9078; CNC. ***Paratypes.*** YEMEN • 1 ♂; same data as holotype; 24 Jun–4 Aug 2003; CD6823; GB: MN182744; CNC.

##### Diagnosis.

This species can be recognized by dark legs; lack of distinct mid-anterior hind tibial bristles and genitalic characters. Due to the shape of the inner male genitalia, it is closely related to *C.conjuncta*, *C.discors*, and *C.mantisphalliga* (*clavata* species group). It differs from these species by the shape of the surstyli which is straight in lateral view and a chitinized lobe in the right gonopod.

##### Description.

**Figure 4.**
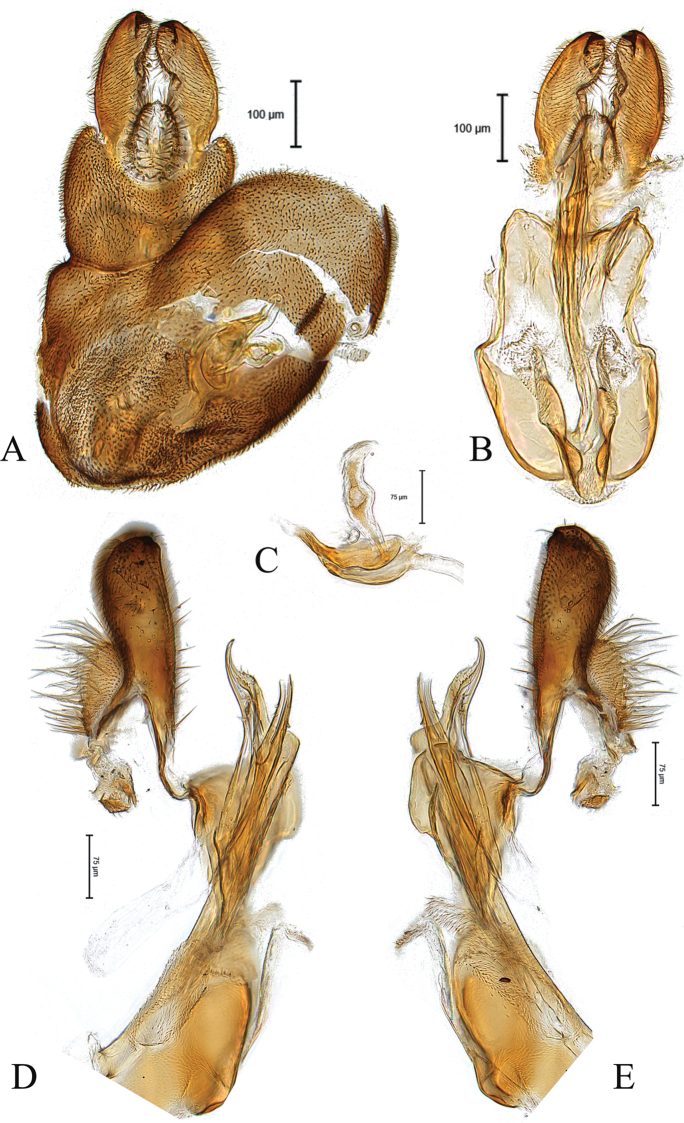
Male genitalia of paratype *Claraeolaheidiae* Motamedinia & Skevington, sp. nov. **A** in dorsal view **B** in ventral view **C** ejaculatory apodeme **D, E** in lateral view.

**Male**. Body length (excluding antennae) 3.1–3.3 mm. ***Head.*** Face dark, silver-gray pruinose. Scape, pedicel, flagellum, and arista dark. Pedicel with three to four dark upper and one long (longer than half of flagellum) and one shorter lower bristle. Flagellum pointed to short tapering (LF:WF = 2.0–2.1) and gray pruinose. Eyes meeting for six or seven times diameter of ocellus. Frons dark, silver-gray pruinose with a weak median keel bearing a shining spot. Vertex dark, lacking pruinosity, bearing an elevated ocellar triangle. Occiput dark, gray pruinose, changing to brown in upper third. ***Thorax.*** Postpronotal lobe brown, gray pruinose and with two to three postpronotal short bristles along upper margin (up to 0.05 mm). Prescutum and scutum dark, predominantly gray-brown pruinose, with two uniseriate dorsocentral rows of dark bristles and longer supra-alar bristles. Scutellum dark, brown pruinose, with a fringe of 10–12 short dark bristles (0.05 mm). Subscutellum large with two gray pruinose patches laterally. ***Wing.*** Length: 3.0–3.2 mm. LW:MWW = 2.4. Wing with microtrichia. Pterostigma light brown and incomplete (LS:LTC = 0.5). LTC:LFC = 1.0. r-m reaches dm between basal third and half of the cell’s length. Halter Length: 0.3 mm. Base and knob dark brown, stem light brown. ***Legs.*** Coxae and trochanters brown, gray pruinose. Mid coxa and hind trochanter with two dark strong bristles on inner apical corner. Hind trochanter with six or seven small black bristles on inner apical corner. Femora dark brown, yellow at apex, with two ventral rows of dark, peg-like spines in apical half. Tibiae dark brown, narrowly pale at base and apices. Hind tibia with two or three wrinkled indentations mid-anteriorly without distinct bristles. Distitarsi brown covered with small black bristles and 1–3 long bristles at apex. Pulvilli slightly smaller than distitarsi. ***Abdomen.*** Ground color dark. Tergite 1 gray pruinose with five or six dark lateral bristles. Tergites 2–5 brown pruinose. Tergite 5 slightly longer than other tergites. Sternites 1–7 dark and pale in middle, gray pruinose. LT35:WS8 = 1.6. Syntergosternite 8 dark brown, gray pruinose, viewed laterally as long as high (LS8:HS8 = 1.0). Viewed caudally, membranous area of medium size and ovate. ***Genitalia.*** Genital capsule dorsal view: epandrium dark, gray pruinose and slightly wider than long (MLE:MWE = 0.8–0.9). Surstyli brown, gray pruinose with somewhat longer bristles, symmetrical, ovate shape and elongated (Fig. 4A). Genital capsule ventral view: gonopods, similar in size, right one with small chitinized lobe and rather large (Fig. 4B). Genital capsule lateral view: surstyli slightly broadened apically (Fig. 4D, E). Phallus strong and straight, with three small ejaculatory ducts, two of them bearing one or two saw-like teeth along their sides (Fig. 4D, E). Phallic guide long, reaching middle of surstyli, with hooked tip (Fig. 4D, E). Ejaculatory apodeme nail-shaped with a bulbous middle (Fig. 4C).

##### Etymology.

The name is selected in honor of Scott Kelso’s daughter for her interest in entomology.

##### Distribution.

Yemen (Fig. 10).

#### Claraeola khorshidae

Taxon classificationAnimaliaDipteraPipunculidae

Motamedinia & Kehlmaier, 2017

06DB087AE9565D3991A7EBE060EE9C6B

Fig. 11

##### Examined material.

***Holotype.*** IRAN • ♂; Southern Khorasan province, Mohammadieh; 32°52'N, 59°01'E; 1419 m; 26 Apr. 2015; Malaise trap; B. Motamedinia leg.; ENA: LT626248; HMIM. ***Paratypes.*** IRAN • 2♂♂; same data as holotype; 23 Aug. 2015; HMIM • 1♀; same data as holotype; 15 Apr. 2015; SMTD; ENA: LT626248 • 2♀♀; same data as holotype; 14 Jul. 2016; HMIM.

##### Diagnosis.

This species can be recognized by distinctly large gonopods, strong bent surstyli in lateral view, and long teeth on three ejaculatory ducts (Fig. 11A–G).

##### Distribution.

Iran (Motamedinia et al. 2017b).

##### Remarks.

This species was described by Motamedinia and Kehlmaier (2017) from the east of Iran.

#### Claraeola khuzestanensis

Taxon classificationAnimaliaDipteraPipunculidae

Motamedinia & Skevington sp. nov.

5FB4131F373C5C47817239E38DE819EF

http://zoobank.org/92df675d-fd91-493a-8a35-bc1390054e84

Figs 5 , 8B , 9E, F

##### Examined material.

***Holotype.*** IRAN • ♂; Khuzestan, Shush; 32°6'N, 48°26'E; 68m; 11 Mar.–10 May 2015; M. Parchami-Araghi leg.; Malaise trap; JSS52300; GB: MN182737; CNC. ***Paratypes.*** IRAN • 1 ♂; 11 Mar.–10 May 2015; E. Gilasian leg.; Malaise trap; JSS52299; GB: 182741; HMIM • 1 ♀; 32°4'N, 48°14'E; same data as holotype; JSS52208; GB: MN182740; CNC • 1 ♀; same data as holotype; 29 Mar.–31 Aug. 2013; JSS52188; GB: MN182743; CNC.

##### Diagnosis.

**Figure 5.**
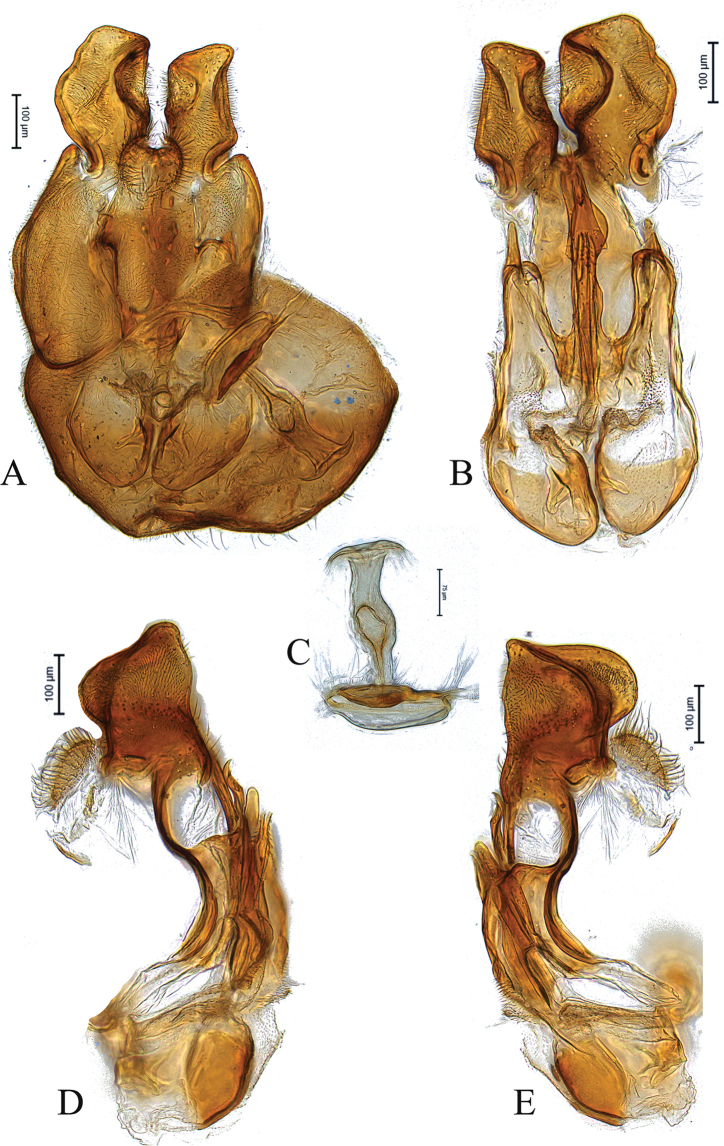
Male genitalia of holotype *Claraeolakhuzestanensis* Motamedinia & Skevington, sp. nov. **A** in dorsal view **B** in ventral view **C** ejaculatory apodeme **D, E** in lateral view.

This species is closely related to *C.parnianae* from southeast Iran, described by Motamedinia and Kehlmaier (2017). Both have a long, slender abdomen with narrow but distinct yellow markings on the abdominal tergites, erect anteromedial setae on mid and hind tibiae and a protruding membranous sheath associated with the ejaculatory ducts. It differs by the shape of surstyli in dorsal view and the structure of the membranous sheath, being trilobate at its apex in *C.parnianae* (Fig. 7A, C, D) It is also closely related to *C.oppleta* (Collin, 1941), recorded from Russia and North Korea and re-described by Kehlmaier (2005b), and *C.alata* (Kozánek & Kwon, 1991), described from North Korea by Kozánek and Kwon (1991). It differs by the square shape of the surstyli in lateral view (Fig. 5D, E), the structure of the phallus with short spines and short membranous sheath without spines (Fig. 5E).

##### Description.

**Male**. Body length (excluding antennae): 4.0–4.3 mm (n = 2). ***Head.*** Face gray pruinose. Scape dark, pedicel partly light brown with four short upper bristles and a long plus a single short lower bristle, flagellum and base of arista completely yellow; flagellum tapering and gray-white pruinose (LF:WF = 3.6). Labellum yellow. Eyes meeting for a distance of 29 facets. Frons silver-gray pruinose. Vertex black, lacking pruinosity. Occiput dark and gray pruinose with scattered small dark bristles. ***Thorax.*** Postpronotal lobe light yellow, gray pruinose. Prescutum black but light brown at lateral margin. Scutum black, gray pruinose with scattered long setae at anterior supra-alar area. Scutellum yellow, with ca. 12 brown setae along posterior margin (up to 0.6 mm). Subscutellum dark, gray pruinose. Pleura dark brown but light brown on anepimeron. ***Wing.*** Length: 3.9–4.1 mm. LW:MWW = 3.1. Wing almost entirely covered with microtrichia. Pterostigma dark-brown and incomplete. LS:LTC = 0.9. LTC:LFC = 1.0. Cross-vein r-m reaches dm shortly after one third of the cell’s length. M_1_ strongly undulating in middle. Halter length: 0.5 mm. Whitish, narrowly brown at base. ***Legs.*** Yellow but brown-yellow at coxae. Coxae gray pruinose. Mid coxa with two or three black anterior bristles. Trochanters partly gray pruinose. Femora gray pruinose. Mid and hind femora bearing two rows of dark, peg-like anteroventral spines in apical one third. Tibiae gray pruinose, with two rows of short setae on anterior and three rows on posterior side. Mid and hind tibiae bearing two or three wrinkled indentations in middle with erect anteromedial setae. Tarsi yellowish but distitarsi brown, with scattered dark setae at anterior margin. Claws white with black tips. ***Abdomen.*** Long and narrow, ground color dark, tergites 1–3 and partly 4 with two narrow posterolateral yellow spots. Tergite 1 gray pruinose, with three or four dark lateral bristles (up to 0.5 mm) and patch of brown setae. Tergites 1–5 with brown setae; tergite 5 posterolaterally gray pruinose. Tergite 5 slightly longer than tergite 4 and almost symmetrical in dorsal view (LT35:WT5 = 1.6, WT5:LT5 = 1.6, T5R:T5L = 1.0). Sternites white-yellow laterally and brown with dark mid-line centrally, gray pruinose. Syntergosternite 8 dark, gray pruinose without dorsal depression on side of right surstylus. LT35:WS8 = 3.0. Viewed laterally, higher than long (LS8:HS8 = 0.7). Membranous area ovate in caudal view, small sized. **Genitalia.** Genital capsule in dorsal view: epandrium and surstyli light brown, gray pruinose. Epandrium wider than long (MLE:MWE = 0.6). Surstyli asymmetrical, right larger than left one. Left surstylus rather rectangular. Right surstylus widened in middle (Fig. 5A). Genital capsule in ventral view: gonopods large and equal in height, humped at apices (Fig. 5B). Genital capsule in lateral view: epandrium without projecting lobe on either side. Surstyli with large projecting lobe on dorsal sides (Fig. 5D, E). Phallus straight, strong, with three short ejaculatory ducts covered with strong and small teeth and a protruding membranous sheath, hook-like at apex (Fig. 5D, E). Phallic guide strong, broaden before narrowed at apex. (Fig. 5D, E). Ejaculatory apodeme tube-like, symmetrical, with a bulb in its middle and fungiform at apex (Fig. 5C).

**Female**. Body length (excluding antennae): 4.1–4.4 mm (n = 2). ***Head.*** Eyes separated, with enlarged frontal facets. Frons gray pruinose in lower half. Occiput gray pruinose. ***Thorax.*** Postpronotal lobe light yellow, gray pruinose with scattered yellow setae. Prescutum and scutum black, gray pruinose with scattered long setae at anterior supra-alar area with two uniseriate dorsocentral rows of hairs. ***Wing.*** Length: 3.8–3.9 mm. LW:MWW = 2.9. Pterostigma light-brown and slightly complete (LS:LTC = 0.8, LTC:LFC = 0.85). ***Legs.*** Yellow, mid coxa and hind trochanter with one or two black anterior bristles. All femora bearing two small ventral rows of dark peg-like spines in the apical third. Hind tibia with two or three wrinkled indentations in middle but without distinctly stronger bristly setae. Basal segment of hind tarsi broad with dense black and yellow bristles. Pulvilli longer than distitarsi. ***Abdomen.*** Tergites with small black bristles. Tergites 1–6 with two narrow posterolateral yellow spots with gray pruinosity. ***Ovipositor.*** Gray pruinose but dorsally with scattered long brown bristles. Viewed laterally: base of piercer curved, piercer slightly angled between proximal and distal part and longer than base (Fig. 8B). LP:LB = 1.9. LDP:LPP = 2.3.

##### Etymology.

Named after Khuzestan, the province from where the holotype originated.

##### Distribution.

Iran (Fig. 10).

#### Claraeola mantisphalliga

Taxon classificationAnimaliaDipteraPipunculidae

Motamedinia & Skevington sp. nov.

DF2CF760D4FF58ABAE39F75F24C87433

http://zoobank.org/64EB602E-60BF-4FF6-B62B-AF28029234BD

Figs 6 , 9B

##### Examined material.

***Holotype.*** YEMEN • ♂; Seyun; 15°57'N, 48°48'E; 20–22 Aug. 2002; light trap; A. van Harten leg.; CD9071; CNC. ***Paratypes.*** YEMEN • 2 ♂♂; same data as holotype; Oct.–Nov. 2002; CD9090; CD9091; CNC.

##### Diagnosis.

This species can be recognized by yellow legs, hind tibia with a wrinkled indentation mid-anteriorly bearing one distinctly stronger bristle and each gonopod with six to seven strong bristles on inner side. It is closely related to *C.conjuncta* and *C.thekkadiensis* (*clavata* species group). It differs by the shape of surstyli in lateral view and the specific shape of the phallic guide with two projecting lobes on either side in ventral view.

##### Description.

**Figure 6.**
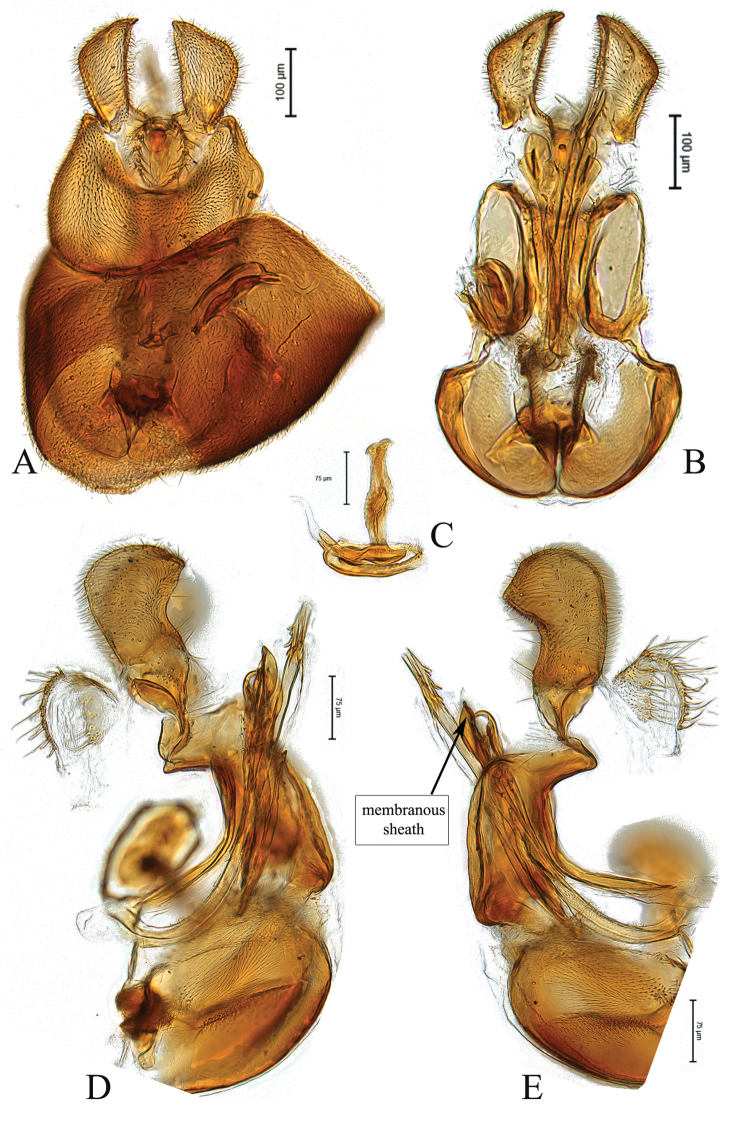
Male genitalia of holotype *Claraeolamantisphalliga* Motamedinia & Skevington, sp. nov. **A** in dorsal view **B** in ventral view **C** ejaculatory apodeme **D, E** in lateral view.

**Male**. Body length (excluding antennae) 3.7–3.8 mm (n = 3). ***Head.*** Face dark, silver-gray pruinose. Scape dark. Pedicel brown, with four dark upper and two lower bristles (the long one is longer than half of flagellum). Flagellum light brown, pointed to tapering (LF:WF = 2.0–2.1) and gray pruinose. Arista dark. Eyes meeting for 11 to 12 times diameter of ocellus. Frons dark, silver-gray pruinose. Vertex dark, lacking pruinosity, bearing an elevated ocellar triangle. Occiput dark, gray pruinose with scattered dark and small bristles. ***Thorax.*** Postpronotal lobe pale, gray pruinose and with three to four postpronotal bristles along upper margin (up to 0.08 mm). Prescutum and scutum dark, predominantly gray pruinose, only dorsocentrally with some brown pruinosity to various extents, with two uniseriate dorsocentral rows of brown bristles and longer supra-alar bristles. Scutellum dark, gray pruinose, with a fringe of 11–13 short dark bristles (up to 0.05 mm). Subscutellum large with two patches of brown-gray pruinose laterally. ***Wing.*** Length: 3.0–3.2 mm. LW:MWW = 2.4. Wing with microtrichia. Pterostigma brown and complete (LS:LTC = 1.0; LTC:LFC = 1). r-m reaches dm between basal third and half of the cell’s length. Halter Length: 0.5 mm and yellow. ***Legs.*** Light brown. Coxae and trochanters gray pruinose. Mid coxa and hind trochanter with one or two dark bristles on inner apical corner. All femora with two ventral rows of dark peg-like spines in apical half. Hind tibia with a wrinkled indentation mid-anteriorly bearing one distinctly stronger bristle. Distitarsi brown covered with small black bristles and three long bristles at apex. Pulvilli slightly longer than distitarsi. ***Abdomen.*** Ground color dark. Tergite 1 gray pruinose with four to five dark lateral bristles. Tergite 2 gray pruinose. Tergites 3–5 laterally gray pruinose, extending onto dorsal surface along posterior margin and largely meeting, otherwise brown pruinose. Sternites 1–7 brown and dark in middle, gray pruinose. LT35:WS8 = 2.3. Syntergosternite 8 dark, gray pruinose, viewed laterally as long as high (LS8:HS8 = 1.0). Viewed caudally, membranous area of medium size, somewhat ovate. ***Genitalia.*** Genital capsule dorsal view: epandrium light brown, gray pruinose and slightly wider than long (MLE:MWE = 0.8–0.9). Surstyli somewhat pale, gray pruinose, symmetrical, slightly bow-shaped and enlarged separation at base (Fig. 6A). Genital capsule ventral view: gonopods equal, rather large, each with six or seven strong bristles on inner side (Fig. 6B). Genital capsule lateral view: phallus with a strong and straight base, with three small ejaculatory ducts, two of them bearing two or three saw-like small teeth along their side (Fig. 6D, E). Phallic guide long and broad, especially at apex with two projecting lobes on either side with hooked tip (Fig. 6D, E). Epandrium with small projecting lobe on either side. Surstyli broadened apically (Fig. 6D, E). Ejaculatory apodeme nail-shaped with a bulbous middle (Fig. 6C).

##### Etymology.

From *mantis* (common name of Mantidae family) and *phalliga* (phallic guide), referring to the similarity between the shape of the phallic guide and a mantis in ventral view.

##### Distribution.

Yemen (Fig. 10).

#### Claraeola parnianae

Taxon classificationAnimaliaDipteraPipunculidae

Motamedinia & Kehlmaier, 2017

DDA1E598206253A4AB67115BDC0E5467

Figs 7 , 8C

##### Examined material.

IRAN • 3♂♂; Sistan & Balochestan, Zabol, Sadesistan; 31°5'N, 61°26'E; 485m; 14 Apr. 2015; H. Derafshan leg.; Malaise trap; JSS51911; GB: MN182735; JSS51912, JSS51913 • 1♀; JSS51910; GB: MN182736; all CNC.

##### Diagnosis.

**Figure 7.**
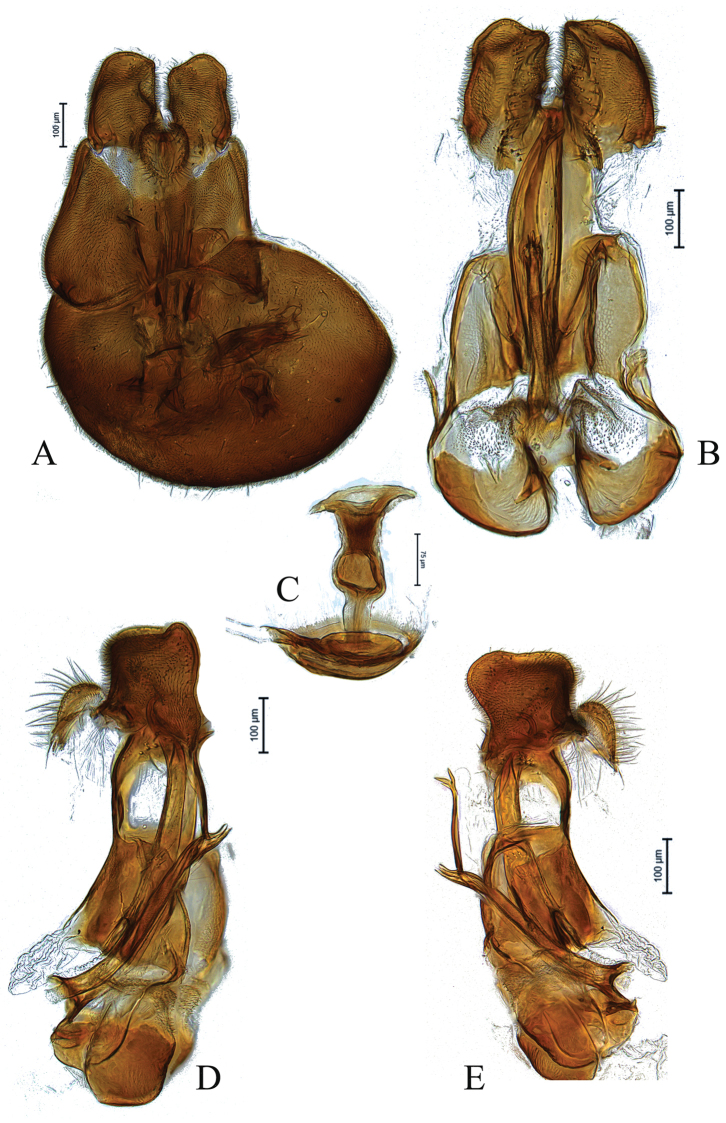
Male genitalia of *Claraeolaparnianae* Motamedinia & Kehlmaier **A** in dorsal view **B** in ventral view **C** ejaculatory apodeme **D, E** in lateral view.

This species stands closely to *C.oppleta* (Collin) and *C.alata* (Kozánek & Kwon). It can be identified by the narrow but distinct yellow markings on the abdominal tergites; erect anteromedial setae on hind tibia; the lateral shape of the surstyli and the trilobate structure of membranous sheath at its apex (Fig. 7; Motamedinia et al. 2017b: fig. 3B).

##### Distribution.

Iran (Fig. 10) (Motamedinia et al. 2017b).

##### Remarks.

This species was described by Motamedinia and Kehlmaier (2017) from southeast Iran.

**Figure 8.**
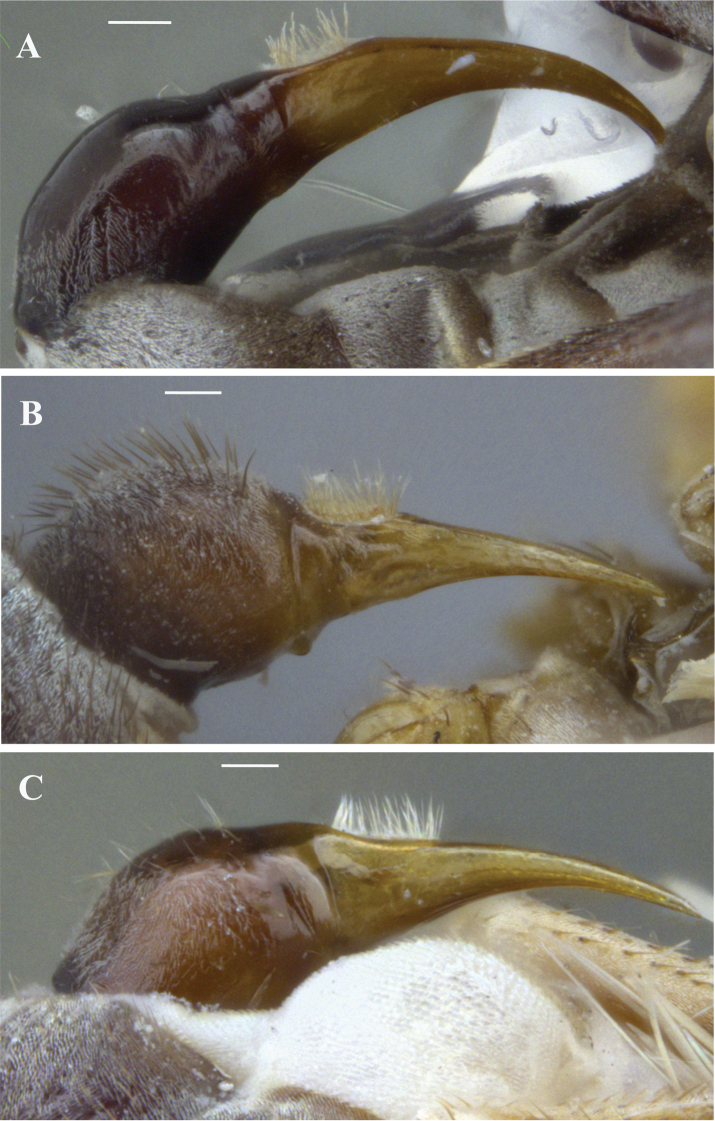
Ovipositor in lateral view **A***Claraeolabousynterga* Motamedinia & Skevington, sp. nov. **B***Claraeolakhuzestanensis* Motamedinia & Skevington, sp. nov. **C***Claraeolaparnianae* Motamedinia & Kehlmaier. Scale bar: 0.1 mm (**A–C**).

#### Claraeola thekkadiensis

Taxon classificationAnimaliaDipteraPipunculidae

(Kapoor, Grewal & Sharma, 1987) comb. nov.

6B76ECB3299A5B139AA871F8F86D76F9

Eudorylas thekkadiensis Kapoor, Grewal & Sharma, 1987:111

##### Examined materal.

***Holotype.*** • ♂; Thekkady (Kerala); 24 Feb. 2019; J.S. Grewal; ***Allotype.*** • 1♀; same data as holotype; S.K. Sharma. ***Paratype.*** • 2♂♂; same data as holotype • 3♂♂; Ramgrah (Bihar); 22 Mar. 1958; S.K. Sharma • 2♀♀; Ranchi (Bihar); 22 Mar. 1985; V.K. Kohli • 1♂; Ranikhet (U.P.); 8 Oct. 1985; S.K. Sharma; Depository: all INPC.

##### Distribution.

India.

##### Remarks.

Although not a Middle Eastern species, this is similar to *C.mantisphalliga* so relevant to this paper. From the detailed drawings of the male genitalia included in the original description (Kapoor et al. 1987), the taxon is transferred from *Eudorylas* to the *Claraeola*.

**Figure 9.**
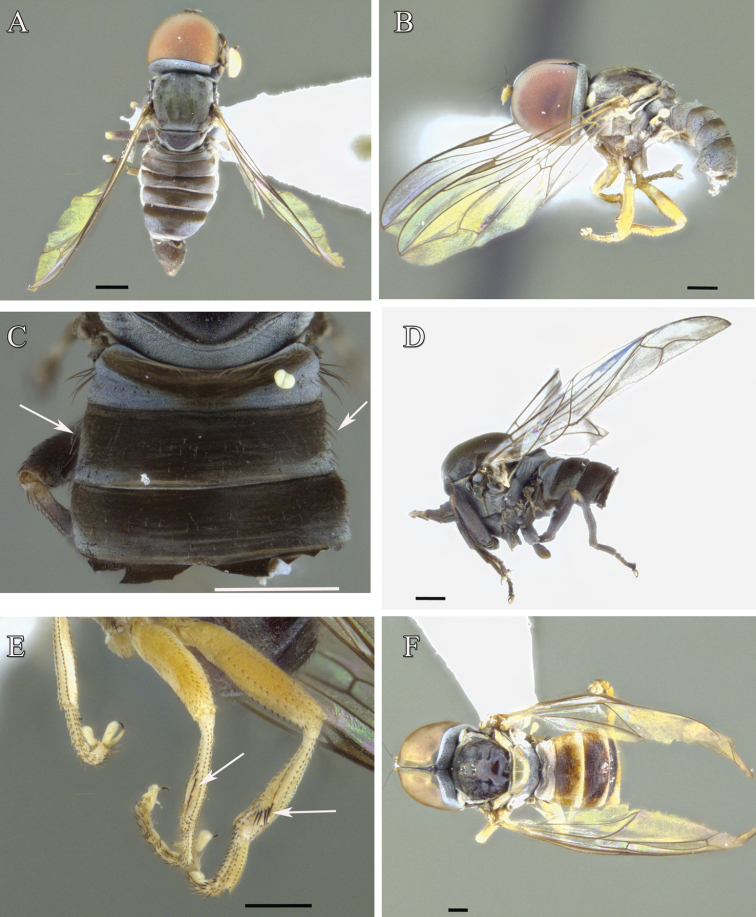
Male of *Claraeola* species **A** paratype of *Claraeolabousynterga* Motamedinia & Skevington, sp. nov. in dorsal view **B** holotype of *Claraeolamantisphalliga* Motamedinia & Skevington, sp. nov. in lateral view **C–D***Claraeolahalterata* (Meigen) **C** abdominal tergites in dorsal view **D** habitus in lateral view **E–F** paratype of *Claraeolakhuzestanensis* Motamedinia & Skevington, sp. nov. **E** legs in lateral view **F** habitus in dorsal view. Scale bar: 0.5 mm (**A–F**).

**Figure 10.**
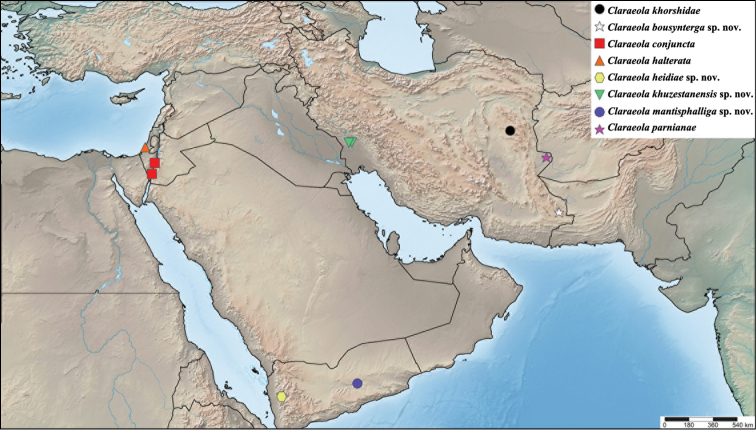
*Claraeola* species distribution in the Middle East.

**Figure 11.**
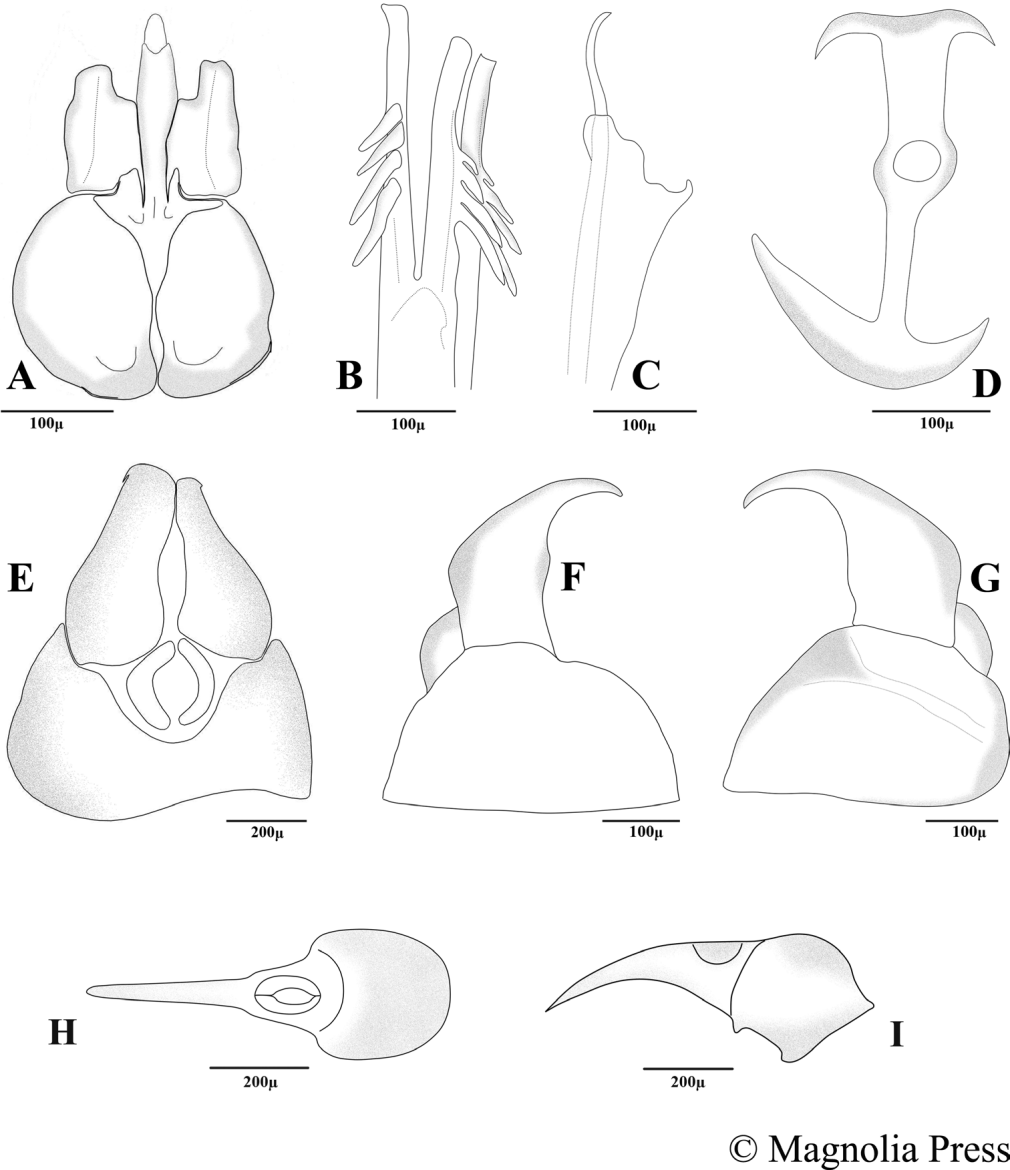
Terminalia of male (**A–G**) and female (**H–I**) of *Claraeolakhorshidae* Motamedinia & Kehlmaier **A** phallic guide, gonopods and hypandrium in ventral view **B** distiphallus with ejaculatory ducts in lateral view **C** phallic guide in lateral view **D** ejaculatory apodeme **E** surstyli in dorsal view **F** left surstylus in lateral view **G** right surstylus in lateral view in ventral view **H** ovipositor in dorsal view **I** ovipositor in lateral view (plate reproduced with permission from copyright holder).

### DNA barcoding

Pipunculidae is a taxonomically challenging family as are most parasitoid taxa. Many are small, most characters are related to male genitalia and many are subtle or difficult to interpret, sexes are difficult to associate, and females are character-poor. For this reason, incorporating both morphological and DNA-sequence data, such as COI DNA barcodes, is critical for species recognition. Based on morphology and DNA barcoding, the present paper introduces four new species of *Claraeola* and associated males and females of two of the new species, *C.bousynterga* sp. nov. and *C.khuzestanensis* sp. nov. DNA sequence data are provided for six Middle Eastern pipunculid species.

Interspecific genetic distances within the Middle Eastern *Claraeola* range from 8.7% (*C.khorshidae* to *C.heidiae*) to 20.6% (*C.halterata* to *C.conjuncta* and *C.heidiae*), while intraspecific genetic distances range from 0% (in *C.khuzestanensis* and *C.parnianae*) to 1.2% (*C.heidiae*). Based on uncorrected pairwise genetic distances (p-distance), *C.heidiae* sp. nov., *C.bousynterga* sp. nov., and *C.conjuncta*, are close to *C.khorshidae* (LT626248), differing by 8.75%, 12.5% and 12.5% respectively. *Claraeolaparnianae* is most similar to *C.khuzestanensis* sp. nov. differing by 9.38% (Table 2).

**Table 2.** Uncorrected pairwise distances among *Claraeola* species in the Middle East (intraspecific distances are highlighted in bold).

		1	2	3	4	5	6	7	8	9	10	11	12	13
1	*C.heidiae*-CD6823*(C)													
2	*C.heidiae*-CD9078*(C)	**0.012**												
3	*C.conjuncta*-JSS50784*(C)	0.118	0.125											
4	*C.halterata*-JSS51645*(ABC)	0.206	0.206	0.162										
5	*C.bousynterga*-JSS51829	0.168	0.168	0.150	0.168									
6	*C.parnianae*-JSS51910	0.162	0.162	0.137	0.112	0.137								
7	*C.parnianae*-JSS51911	0.162	0.162	0.137	0.112	0.137	**0.000**							
8	*C.bousynterga*-JSS51920*(C)	0.162	0.162	0.143	0.162	**0.006**	0.137	0.137						
9	*C.bousynterga*- JSS52173	0.168	0.168	0.150	0.168	**0.000**	0.137	0.137	**0.006**					
10	*C.khuzestanensis*-JSS52188	0.137	0.137	0.143	0.143	0.137	0.093	0.093	0.131	0.137				
11	*C.khuzestanensis*-JSS52208	0.143	0.143	0.150	0.150	0.143	0.100	0.100	0.137	0.143	**0.006**			
12	*C.khuzestanensis*-JSS52299*(C)	0.137	0.137	0.143	0.143	0.137	0.093	0.093	0.131	0.137	**0.000**	**0.006**		
13	*C.khuzestanensis*-JSS52300	0.137	0.137	0.143	0.143	0.137	0.093	0.093	0.131	0.137	**0.000**	**0.006**	**0.000**	
14	*C.khorshidae*-LT626248	0.087	0.087	0.125	0.168	0.131	0.118	0.118	0.125	0.131	0.093	0.100	0.093	0.093

* Specimen sequence data was obtained using the COI mini-barcode protocol. A, B, & C denote the COI mini-barcode regions sequenced.

## Supplementary Material

XML Treatment for Claraeola

XML Treatment for Claraeola bousynterga

XML Treatment for Claraeola conjuncta

XML Treatment for Claraeola halterata

XML Treatment for Claraeola heidiae

XML Treatment for Claraeola khorshidae

XML Treatment for Claraeola khuzestanensis

XML Treatment for Claraeola mantisphalliga

XML Treatment for Claraeola parnianae

XML Treatment for Claraeola thekkadiensis

## References

[B1] Aczél ML (1940) Vorarbeiten zu einer Monographie der Dorylaiden (Dipt.). Dorylaiden-Studien V. Zoologischer Anzeiger 132: 149–169.

[B2] Földvári M (2013) Taxonomic revision of the Afrotropical species of the tribe Eudorylini (Diptera, Pipunculidae). Zootaxa 3656, 1–121. 10.11646/zootaxa.3656.1.1 25333089

[B3] Folmer O Black M Hoeh W Lutz R Vrijenhoek R (1994) DNA primers for amplification of mitochondrial cytochrome c oxidase subunit I from diverse metazoan invertebrates. Moelecular Marine Biology and Biotechnology 3: 294–299. 7881515

[B4] Gibson JF Kelso S Jackson MD Kits JH Miranda GFG Skevington J (2011) Diptera-Specific Polymerase Chain Reaction Amplification Primers of Use in Molecular Phylogenetic Research. Annals of the Entomological Society of America 104: 976–997. 10.1603/AN10153

[B5] Hardy DE (1964) Family Pipunculidae Zetterstedt. The big-headed or big-eyed flies. In: Zimmerman EC (Ed.) Insects of Hawaii.University of Hawaii Press, Honolulu, 302–379.

[B6] Jervis MA (1992) A taxonomic revision of the pipunculid fly genus *Chalarus* Walker, with particular reference to the European fauna. Zoological Journal of the Linnean Society 105: 243–352. 10.1111/j.1096-3642.1992.tb01232.x

[B7] Kapoor VC Grewal JS Sharma SK (1987) Indian Pipunculids (Diptera: Pipunculidae). A Comprehensive Monograph. Atlantic Publishers & Distributors, New Delhi, 201 pp.

[B8] Kehlmaier C (2005a) Taxonomic revision of European Eudorylini (Insecta, Diptera, Pipunculidae). Verhandlungen des Naturwissenschaftlichen Vereins in Hamburg, Neue Folge 41: 45–353.

[B9] Kehlmaier C (2005b) Taxonomic studies on Palaearctic and Oriental Eudorylini (Diptera: Pipunculidae), with the description of three new species. Zootaxa 1030: 1–48. 10.11646/zootaxa.1030.1.1

[B10] Kehlmaier C Dierick M Skevington JH (2014) Micro-CT studies of amber inclusions reveal internal genitalic features of big-headed flies, enabling a systematic placement of *Metanephrocerus* Aczél, 1948 (Insecta: Diptera: Pipunculidae). Arthropod Systematics and Phylogeny 72: 23–36.

[B11] Kehlmaier C Gibbs D Withers P (2019) New records of big-headed flies (Diptera: Pipunculidae) from the Mediterranean Basin. Bonn zoological Bulletin 68(1): 31–60.

[B12] Koenig DP Young CW (2007) First observation of parasitic relations between big-headed flies, *Nephrocerus* Zetterstedt (Diptera: Pipunculidae) and crane flies, *Tipula* Linnaeus (Diptera: Tupulidae: Tipulinae), with larval and puparial descriptions for the genus *Nephrocerus*. Proceedings of the Entomological Society of Washington 109: 52–65.

[B13] Kozánek M Kwon YJ (1991) Classification of the Family Pipunculidae from Korea (Diptera) Part I. On the genus *Moriparia* gen.nov. from North Korea. Insecta Koreana 8: 76–84.

[B14] Kozánek M Suh SJ Kwon YJ (2003) Taxonomy of the genus *Moriparia* Kozanek et Kwon (Diptera: Pipunculidae) from Korea. Korean Journal of Entomology 33: 99–103. 10.1111/j.1748-5967.2003.tb00057.x

[B15] Kumar S Stecher G Tamura K (2016) MEGA7: Molecular Evolutionary Genetics Analysis version 7.0 for bigger datasets. Molecular Biology and Evolution 33(7): 1870–1874. 10.1093/molbev/msw054 27004904 PMC8210823

[B16] Littlefield R (2018) Zerene Stacker. http://zerenesystems.com/cms/stacker

[B17] Maddison WP Maddison DR (2018) Mesquite: a modular system for evolutionary analysis. Version 3.51. http://www.mesquiteproject.org

[B18] Motamedinia B Mokhtari A Rakhshani E Gilasian E (2017a) Review of Eudorylini (Diptera, Pipunculidae, Pipunculinae) from Iran with four new species records. Journal of Insect Biodiversity and Systematics 03(4): 335–346.

[B19] Motamedinia B Kehlmaier C Mokhtari A Rakhshani E Gilasian E (2017b) Discovery of the genus *Claraeola* Aczél in Iran with the description of two new species (Diptera: Pipunculidae). Zootaxa 4227: 563–572. 10.11646/zootaxa.4227.4.6 28187567

[B20] Rafael JA De Meyer M (1992) Generic classification of the family Pipunculidae (Diptera): a cladistic analysis. Journal of Natural History 26: 637–658. 10.1080/00222939200770391

[B21] Rafael JA Skevington JH (2010) Pipunculidae (Big-headed Flies). In: Brown BV Borkent A Cumming JM Wood DM Woodley NE Zumbado MA (Eds) Manual of Central American Diptera.INBio, San Jose, 793–803.

[B22] Shorthouse (2010) SimpleMappr, an online tool to produce publication-quality point maps. http://www.simplemappr.net [accessed 20 April 2012.2012]

[B23] Skevington JH (2002) Phylogenetic revision of Australian members of the *Allomethus* genus group (Diptera: Pipunculidae). Insect Systematics and Evolution 33: 133–161. 10.1163/187631202X00109

[B24] Skevington JH (2019) Catalogue of the big-headed flies (Diptera, Pipunculidae) of the World. [Unpublished database]

[B25] Skevington JH Marshall SA (1997) First record of a big-headed fly, *Eudorylasalternatus* (Cresson) (Diptera: Pipunculidae), reared from the subfamily Cicadellinae (Homoptera: Cicadellidae), with an overview of pipunculid-host associations in the Nearctic Region. The Canadian Entomologist 129: 387–398. 10.4039/Ent129387-3

[B26] Skevington JH Yeates DK (2001) Phylogenetic classification of Eudorylini (Diptera: Pipunculidae). Systematic Entomology 26: 421–452. 10.1046/j.0307-6970.2001.00160.x

[B27] Wada S (1991) Morphologische Indizien für das unmittelbare Schwestergruppenverhältnis der Schizophora mit den Syrphoidea (‘Aschiza’) in der phylogenetischen Systematik der Cyclorrhapha (Diptera: Brachycera). Journal of Natural History 25: 1531–1570. [Morphological evidence for the direct sister group relationship between the Schizophora and the Syrphoidea (Aschiza) in the phylogenetic systematics of the Cyclorrhapha (Diptera: Brachycera)] 10.1080/00222939100770971

[B28] Weintraub PG Beanland L (2006) Insect Vectors of Phytoplasmas. Annual Review of Entomology 51: 91–111. 10.1146/annurev.ento.51.110104.151039 16332205

